# PReS-FINAL-2025: Arthritis associated with human immunodeficiency virus

**DOI:** 10.1186/1546-0096-11-S2-P38

**Published:** 2013-12-05

**Authors:** AE Bean, M Al-Obaidi, F Shakley, C Waruiru, D Hawley

**Affiliations:** 1 Paediatric Rheumatology, Sheffield Children's Hospital, Sheffield, UK; 2 Paediatric Infectious Disease and Immunology, Sheffield Chidren's Hospital, Sheffield, UK

## Introduction

There is a clear, documented association between Human Immunodeficiency Virus (HIV) and arthritis in children and young people (CYP). However, this association has not been clearly defined and the arthritis has been seen to resolve with differing management strategies.

## Objectives

- To present a case of a child diagnosed and treated for Juvenile Idiopathic Arthritis (JIA) who was subsequently found to have HIV which led to treatment modification.

- To discuss HIV testing in CYP presenting with arthritis.

- To explore appropriate treatment options in CYP with HIV who have arthritis.

## Methods

We retrospectively reviewed the case record of a fourteen year old boy of African origin with arthritis. He had been previously well and presented with a two week history of initially right ankle swelling which progressed to involve his left knee, right ankle, left wrist and distal interphalangeal joint of his index finger of his left hand. All these joints were swollen, warm and had restricted range of movement. He was systemically well and was diagnosed with polyarticular JIA. Initial management consisted of oral Naproxen, Prednisolone 0.6 mg/kg and Methotrexate 15 mg/m^2^.

This treatment resulted in some improvment in his joint symptoms. However, he represented two weeks later experiencing night sweats and was found to have generalised lymphadenopathy. After further history and investigation, he was diagnosed with HIV infection. He was subsequently referred to tertiary centre specialists in Paediatric Rheumatology and Infectious Diseases/Immunology (at Sheffield Children's Hospital) for further investigation.

## Results

His CD4 count was 295 × 10^6^/L and viral load of 8487 copies/ml. Extensive investigation did not reveal another infective cause for his joint symptoms. At presentation his C reactive protein (CRP) was 18 mg/L and erythrocyte sedimentation rate (ESR) >145 mm/hr. Anti-nuclear antibodies and extractable nuclear antigens were negative; double stranded DNA was 160 IU/ml.

Within a multidisciplinary team including specialists in Infectious Diseases and Rheumatology, the current literature was reviewed. He was managed with intra-articular administration of Triamcinolone Hexacetonide into affected joints, and antiretroviral therapy. Two weeks following this he was re-assessed showing complete resolution of his joint signs and symptoms. Inflammatory markers normalised with CRP <7 mg/L and ESR 5 mm/hr.

## Conclusion

This case raises awareness of arthritis as a presenting feature of HIV in CYP. Furthermore, it raises the question of which CYP with JIA should be tested for HIV. It also highlights that more international collaborative work is required to determine optimal treatment strategies for HIV associated arthritis in CYP.

## Disclosure of interest

None declared.

